# Same agent, different messages: insight into transcriptional regulation by SIN3 isoforms

**DOI:** 10.1186/s13072-018-0188-y

**Published:** 2018-04-17

**Authors:** Ashlesha Chaubal, Lori A. Pile

**Affiliations:** Department of Biological Sciences, Wayne State University, Detroit, MI 48202 USA

**Keywords:** SIN3 isoforms, Transcription, Chromatin, Posttranslational modification

## Abstract

SIN3 is a global transcriptional coregulator that governs expression of a large repertoire of gene targets. It is an important player in gene regulation, which can repress or activate diverse gene targets in a context-dependent manner. SIN3 is required for several vital biological processes such as cell proliferation, energy metabolism, organ development, and cellular senescence. The functional flexibility of SIN3 arises from its ability to interact with a large variety of partners through protein interaction domains that are conserved across species, ranging from yeast to mammals. Several isoforms of SIN3 are present in these different species that can perform common and specialized functions through interactions with distinct enzymes and DNA-binding partners. Although SIN3 has been well studied due to its wide-ranging functions and highly conserved interaction domains, precise roles of individual SIN3 isoforms have received less attention. In this review, we discuss the differences in structure and function of distinct SIN3 isoforms and provide possible avenues to understand the complete picture of regulation by SIN3.

## Background

The basic requirement for survival of any cell, whether prokaryotic or eukaryotic, is the accurate expression of appropriate genes. In prokaryotes, failure to express important metabolic enzyme or stress response genes can affect the organism’s ability to respond to environmental stimuli and ultimately result in cell death. In multicellular organisms, regulation of gene expression is critical not only for response to external stimuli but also to ensure timely expression of cell type-specific genes for the development of different tissues and organs. The presence of chromatin, a complex of the DNA molecule wrapped around a core of histone proteins, is key for gene regulation in eukaryotes.

Precise regulation of spatiotemporal gene expression is orchestrated by the action of many vital players that govern the organization and compaction of chromatin. Chromatin-modifying complexes can determine whether the chromatin is compact and therefore refractory to transcription factor binding, or is more accessible, thereby amenable to gene expression. Factors that regulate the level of acetylation of histone proteins are an excellent example of this phenomenon. Enzymes that acetylate specific amino acid residues on N-terminal tails of histone proteins, histone acetyltransferases (HATs), can activate gene transcription, whereas enzymes that remove the acetyl mark from these residues, histone deacetylases (HDACs), typically promote gene silencing. The SIN3 histone-modifying complex is one such important player that regulates various biological processes through activation or repression of a large repertoire of target genes. SIN3 was first discovered in 1987 by two independent research groups studying mating type switching in *Saccharomyces cerevisiae* [1, 2]. Both groups identified SIN3 as a negative regulator of the HO (homothallic switching) endonuclease, which is essential for mating type switching in yeast. In the decade following its discovery, SIN3 was identified in independent genetic screens under five different aliases, *UME4*, *RPD1*, *GAM2*, *CPE1*, and *SDS16*, primarily as a negative regulator of transcription [3–7]. In 1997, three separate studies showed that SIN3 is associated with the histone deacetylases HDAC1/2 in a multi-protein complex [8–10]. As deacetylated histones have long been correlated with transcription inactivity, the SIN3 complex is canonically regarded as a corepressor complex [11].

Accumulating evidence, however, points to a dual role of the SIN3 complex in the regulation of transcription (Table 1). The transcriptional profile of a *SIN3* deletion yeast strain showed upregulation of 173 transcripts confirming the role of Sin3 in gene repression [12]. In addition, 269 transcripts were downregulated in the absence of Sin3, suggesting a possible role in gene activation. A genome-wide study performed using a *Drosophila* cell culture system comparing wild type and *Sin3A* RNA interference-mediated knockdown cells showed a similar result to that of yeast mutants [13]. Out of the 13,137 genes that were tested by microarray analysis, SIN3 was required for the repression of 364 genes, whereas 35 genes were activated by SIN3. Further evidence for the dual role of SIN3 came from a gene expression analysis in another important model system. Loss of SIN3 in mouse fibroblast cells resulted in differential expression of 1308 genes, out of which 977 were upregulated and 331 were downregulated [14].

**Table 1:** SIN3 plays a dual role in transcription regulation

Organism	Isoform studied	Transcriptome analysis		Genome-wide binding		GO categories	Ref
		Genes activated	Genes repressed	Genes activated	Genes repressed		
*S. c.*	na	269	173	ns		Cell cycle regulation, carbon metabolite, carbohydrate utilization, and transport	[12]^#^
	na	ns		100		Cell cycle regulation	[69]*
*D. m.*	SIN3 220	35	364	ns		Cell cycle regulation, signal transduction, transcription, metabolism	[13]^#^
	SIN3 187	605	669	338	282	Both: cell cycle regulation, intracellular transport, metabolism, SIN3 187: development, apoptosis	[20]^##^**
	SIN3 220	349	263	162	243		
*M. m.*	SIN3A	331	977	ns		Cell cycle regulation, DNA repair, energy generation	[14]^#^
	SIN3A	ns		707		Cell cycle regulation, metabolism, transcription	[53] *
	SIN3B			686			
	SIN3A	ns		1491		*Both:* cell cycle regulation, RNA processing, DNA replication/repair *SIN3B* redox, protein transport, protein catabolism	[61] **
	SIN3B			4993			
*H. s.*	SIN3A	2587	2662	1120	1586	Cellular process, development	[19]^#^**

Genome-wide expression and binding analyses for SIN3 in different organisms. Enriched GO categories are listed to indicate biological functions of regulated genes

*Na,* not applicable; *ns* not studied. *S.c., Saccharomyces cerevisiae*; *D.m., Drosophila melanogaster; M.m., Mus musculus; H.s., Homo sapiens*

Asterisk and hashtag symbols indicate the experimental approach used in specific studies to generate expression and binding data

^#^Microarray, ^##^RNA-seq, *ChIP-chip, **ChIP-seq

Although the initial transcriptome studies revealed several gene targets that were downregulated upon loss of SIN3, the role of SIN3 in gene activation was not well understood and was commonly attributed to indirect effects. Activation of transcription by SIN3 could conceivably result from a secondary effect; yet, several gene-specific studies suggest otherwise. NANOG is a transcription factor critical for maintaining the pluripotent state in embryonic stem cells [15]. During embryonic stem cell differentiation, phosphorylated p53 suppresses *Nanog* expression by recruiting SIN3A to the *Nanog* promoter [16]. Conversely, under proliferating conditions, the SIN3A/HDAC complex is recruited to the *Nanog* promoter leading to Sox2-mediated stimulation of *Nanog* expression [17]. Thus, SIN3A regulates *Nanog* expression either positively or negatively, in a context-dependent manner. SIN3A also plays a dual role in regulation of STAT transcriptional activity [18]. STAT1 and STAT3 perform opposing functions in the regulation of cell proliferation and survival. SIN3A interacts with STAT3 and acts as a repressor of STAT3 activity. In contrast, SIN3A is required for the transcription of ISGF3 (STAT1:STAT2:IRF9) complex-regulated genes. Studies performed in human kidney embryonic cells and *Drosophila* S2 cells provide a clearer picture of the direct role of SIN3 in gene transcription, by integrating transcriptome data with genome-wide binding data (Table 1). For human SIN3A, 42% of activated genes and 61% of repressed genes were directly bound by the corepressor [19]. In *Drosophila* S2 cells, 92% of genes repressed and 46% of genes activated by SIN3 were also bound by SIN3, further highlighting SIN3 as a dual regulator of transcription [20].

The corepressor activity of SIN3 is commonly attributed to its association with the deacetylase enzymes HDAC1 and HDAC2. The ways in which the complex may function to activate gene transcription, however, are not understood. There are a number of potential molecular mechanisms in which SIN3 complex activity could lead to gene activation. One possibility is that the HDAC1 component of the complex deacetylates a DNA-binding transcription factor, which alters the DNA-binding capability or protein interactions of the transcription factor. Two examples of transcription factors demonstrated to be regulated by acetylation are YYI, which can function as both a repressor and as an activator, and Foxo1 [21, 22]. The full list of possible transcription factor substrates for HDAC1 has yet to be elucidated. A second possible mechanism in which the SIN3 complex could activate gene transcription is by influencing nucleosome occupancy around transcription start sites. Chen et al. [23] found that yeast Rpd3-containing complexes have chaperone activity and impact nucleosome assembly in vitro. The data presented in that work principally focused on the role of the complex in repression. In their discussion, however, the authors speculate that nucleosome remodeling could either facilitate or repress transcription of gene targets. In addition to histone modification, DNA methylation influences transcription activity. SIN3 interacts with the DNA demethylase TET1, and the two proteins colocalize at a number of genomic sites [19]. It is possible that SIN3 complex association with TET1 impacts local DNA methylation levels, which then leads to transcription activation. Much work remains for the future to determine the specific mechanisms in which a single complex can have opposite effects.

**Fig. 1:**
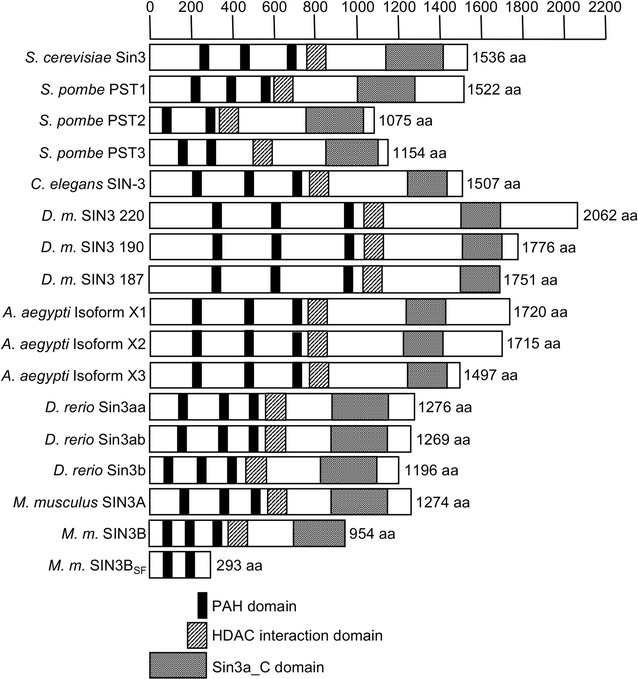
Schematic representation of SIN3 isoforms in different species. This schematic was generated using the batch-CD (conserved domain) search interface from NCBI [67, 68]. The scale at the top represents amino acid positions in the protein. PAH domains are paired amphipathic helix domains involved in protein–protein interaction. HDAC interaction domain (HID), also known as Sin3 family corepressor domain, is involved in interaction with HDACs. Sin3a_C domain is a conserved domain found in the SIN3 protein family at the C-terminus, downstream of the Sin3 family corepressor domain. This region contains a protein–protein interaction domain and a region of high sequence conservation with unknown function, previously known as PAH4 and HCR, respectively. *D.m., Drosophila melanogaster, M.m., Mus musculus*

The ability of SIN3 to repress or activate gene transcription is likely due to its interaction with a large repertoire of DNA-binding factors. The SIN3 protein contains six highly conserved regions (Fig. 1). These were initially described as four paired amphipathic alpha-helix motifs (PAH1-4), a histone deacetylase interaction domain (HID) and a highly conserved region (HCR) [24, 25]. Recent updates in terminology refer to the region containing the PAH4 domain and the HCR as the Sin3a_C superfamily domain (Fig. 1). These domains, conserved from yeast to mammals, are essential for interaction with the core components of the SIN3 complex and other interacting partners that recruit the complex to its target genes. Due to the presence of these protein–protein interaction domains, SIN3 is believed to be the scaffold that holds the complex together. Co-immunoprecipitation studies performed by different research groups showed that the SIN3 core complex consists of HDAC1, HDAC2, RbAp46/48, SAP30, SAP18, and SDS3 [8–10, 26]. Over the years, a multitude of proteins including SAP130 and SAP180, ING1/2, RBP1, FAM60A, BRMS1, Pf1, KDM5A/B, and MRG15 have been reported to interact with the SIN3 complex suggesting that several SIN3 sub-complexes may exist [27–29].

The variable interactions, with a diversity of accessory factors and distinct enzymatic modules, contribute to the functional flexibility of the SIN3 complex. It is important to note that multiple isoforms of SIN3 and other complex components exist, which likely adds to the modularity of the SIN3 complex. Several studies, described below, provide evidence that SIN3 isoforms perform non-redundant functions despite the presence of highly conserved protein interaction domains. This review focuses on understanding the structural and functional differences of SIN3 isoforms.

## SIN3 isoform conservation across species

In budding yeast, *Saccharomyces cerevisiae*, there is a single *SIN3* gene that gives rise to a single acidic protein of approximately 170 kDa, which contains the PAH and HID domains mentioned above important for protein–protein interactions (Fig. 1). In *Schizosaccharomyces pombe*, there are three distinct *Sin3* genes, *pst1*, *pst2,* and *pst3*, encoding proteins that contain PAH and HID domains and that exhibit high levels of conservation with the *Saccharomyces cerevisiae* Sin3 [24, 30]. Unlike fission yeast, the nematode *Caenorhabditis elegans* has only one *Sin3* gene that gives rise to a protein containing a single PAH domain and a HID region [31]. *Drosophila* also has a single *Sin3A* gene, encoding a larger SIN3 protein as compared to the yeast and worm proteins [32]. The *Drosophila Sin3A* gene produces three alternatively spliced isoforms that differ at their C-terminus [33, 34] (Fig. 1). These isoforms are referred to as SIN3 220, SIN3 190, and SIN3 187, based on their molecular weights. All three isoforms possess the protein interaction domains, PAH1-3 and HID, but have unique stretches of amino acids at the C-terminus. SIN3 220 has 315, SIN3 190 has 31, and SIN3 187 has five unique amino acids. Intriguingly, there is interplay between the predominant SIN3 isoforms, SIN3 220 and SIN3 187, wherein overexpression of the lower molecular weight isoform, SIN3 187, causes a reduction in transcript and accelerated proteasomal degradation of endogenous SIN3 220 [35]. It is possible that similar processes are at work in other organisms, considering the high degree of evolutionary conservation for SIN3 and its roles across species.

In the vertebrate lineage, a duplication event gave rise to two *Sin3* genes, *Sin3a* and *Sin3b*. In zebrafish, however, there are three *Sin3* genes, *sin3aa*, *sin3ab,* and *sin3b,* as a result of a second gene duplication event specific to teleost fish [36]. The paralogs Sin3aa and Sin3ab are 78% identical to each other and 50% identical to their ortholog Sin3b, exhibiting high levels of similarity between the PAH and HID domains. Mammalian cells contain two *Sin3* genes, *Sin3a* and *Sin3b* [37]. The murine SIN3A and SIN3B proteins are highly similar throughout their length, with highest homology at the PAH and HID domains. Compared to SIN3A, SIN3B has a shorter N-terminal region [37]. Multiple variant isoforms of the two mammalian *Sin3* genes have also been reported. The *Sin3a* gene was reported to give rise to at least two alternatively spliced isoforms, SIN3A and SIN3A9, wherein there is a nine-amino acid insert in the SIN3A9 isoform between amino acids 1205 and 1206 relative to SIN3A [37]. The *Sin3b* gene can also undergo alternative splicing [38, 39] (Fig. 1). One splice form of the SIN3B protein is 954 amino acids long and contains the conserved PAH1-3 and HID domains. The alternative form is a 293-amino acid protein, referred to as either SIN3B_SF_ or SIN3B(293), which contains only the PAH1 and PAH2 domains and a unique stretch of 19 amino acids at the C-terminus. The shorter SIN3B isoform does not possess the HID region and therefore does not interact with histone deacetylases, but is still capable of repressing basal transcription [38]. This leads to an intriguing possibility that the SIN3B isoforms may exercise different mechanisms of gene repression. Expression of these isoforms may be regulated, since they can compete for binding partners due to the presence of identical protein interaction domains. It will be interesting to see whether an inter-isoform-dependent regulation of SIN3 as observed in *Drosophila* also occurs in mammalian cells. Furthermore, posttranslational modifications that can target SIN3 isoforms to the proteasome, as described below, may also play an important role in the regulation of SIN3 in a cell type and context-dependent manner.

Degradation by the proteasome often involves SUMO/ubiquitin-mediated protein targeting. Mammalian SIN3 has been found to be a substrate for both sumoylation and ubiquitination. TOPORS (TOP1 binding arginine/serine-rich protein) is a nuclear protein that functions as a RING-dependent E3 ubiquitin ligase and as a SUMO-1 E3 ligase for p53 [40, 41]. SIN3A was identified by mass spectrometry and verified in vitro as a sumoylation substrate of TOPORS in a proteomic screen performed in HeLa cells [42]. SIN3B was not detected in this screen even though other SIN3-associated proteins including RbAp46 and RbAp48 were identified as putative TOPORS substrates. SIN3B was instead identified as a putative target for the E3 ubiquitin ligase RNF220 in a yeast two-hybrid screen [43]. In additional experiments conducted using HEK293 cells, these researchers showed that RNF220 can ubiquitinate the N-terminal PAH1 domain as well as the C-terminus containing PAH3 and Sin3a_C domains of SIN3B and target it for proteasomal degradation. Since SIN3A was not identified in this study, it is probable that RNF220 specifically ubiquitinates SIN3B and not SIN3A. It is conceivable that the SIN3A isoform is sumoylated and the SIN3B isoform is ubiquitinated in a context-dependent manner. Differential posttranslational modifications of SIN3 isoforms could comprise a set of active mechanisms to precisely regulate not only protein level but also the function of SIN3 isoforms in different cell types and during critical biological processes. For example, posttranslational modification of SIN3 could impact the ability of SIN3 to interact with other proteins in the complex or with DNA-binding recruitment factors. The relevance of SIN3 posttranslational modifications in vivo remains to be ascertained, and considering the critical functions of SIN3 in transcription, they may also hold clues into active regulation of other complexes with similar importance. It is noteworthy that the mammalian SIN3 proteins interact with different E3 ligases despite the presence of well-conserved protein–protein interactions domains. The distinct protein interactions, including the E3 ligases as well as other accessory subunits, may be responsible for the non-redundant functions performed by SIN3 isoforms, as discussed below.

## Distinct protein–protein interactions exhibited by SIN3 isoforms

SIN3 proteins serve as the scaffold for a histone-modifying complex [27, 44]. The PAH and HID regions provide interfaces for protein–protein interactions with complex components. The central region of SIN3, which includes the PAH3 and HID domains, interacts with so-named core complex components [44]. SIN3 complexes have been isolated and described in different organisms and distinct cell types [27, 28, 45–47]. Proteins commonly identified in various organisms and found to interact with multiple isoforms include the enzymes HDAC1/2 as well as accessory factors RbAp46/48, ARID4A/4B, PF1, SAP130, SAP180, ING1/2, BRMS1, and SDS3. As described below, certain additional accessory factors, as well as some enzymes, either interact preferentially with a single isoform or have only been found as part of a SIN3 complex in a specific cell type. The N-terminal PAH domains, PAH1 and PAH2, bind to DNA-binding transcription factors that can recruit the SIN3 complex to target genes [48]. Some transcription factors and coregulators interact with multiple isoforms, while others exhibit isoform specificity. A select list of examples is provided in Table 2. Less is known about the interactions mediated by the conserved Sin3a_C domain at the C-terminal region. Despite extensive research that has identified the myriad SIN3 isoform complex components and recruiting factors, specific functions of the different complexes are only beginning to be elucidated.

**Table 2:** SIN3 interacts with a variety of binding partners to perform its biological function

Associated SIN3 isoform	Interacting protein	Function	Ref
SIN3A SIN3B	MAD-MAX	Transcription factors involved in cell proliferation and differentiation	[9, 37]
SIN3A SIN3B	IKAROS	Transcription factor involved in lymphocyte development	[70]
SIN3A SIN3B	FOXK1	Transcription factor involved in regulation of myogenic progenitors	[71]
SIN3A	NANOG	Transcription factor involved in maintaining pluripotency of ES cells	[45, 72]
SIN3A	FAM60A	Transcriptional regulator involved in TGF-β signaling	[28, 46]
SIN3A	MeCP2	Methyl-CpG binding protein	[51]
SIN3A	OGT	O-GlcNac transferase	[73]
SIN3A	TET1	Tet methylcytosine dioxygenase (involved in DNA demethylation)	[19]
SIN3A	BBX	Transcription factor important for cell cycle progression	[74]
SIN3A	SMRT	Hormone-sensitive transcriptional corepressor	[56]
SIN3A	BRG1, hBRM	ATPases involved in chromatin remodeling	[57]
SIN3B	EMSY	Transcriptional corepressor, BRCA2 binding protein	[75]
SIN3B	Na_v_1.2, Na_v_1.6	Voltage-gated sodium channels in neurons	[76]

Representative examples from the SIN3 interactome that contribute to the functional flexibility of SIN3

The highly similar SIN3 isoforms can form different multi-subunit complexes, which in some instances are comprised of distinct histone-modifying enzymes (Table 3). In the fission yeast *Schizosaccharomyces pombe*, the SIN3 proteins Pst1 and Pst2 form distinct complexes that perform non-redundant functions [49]. Pst1 is part of Complex I, which contains Clr6, Prw1, and Sds3 and regulates histone acetylation at promoter regions. Pst2 is associated with Complex II that includes Clr6, Prw1, Alp13, Cph1, and Cph2. Complex II primarily deacetylates histones in gene coding regions. Although there is a single Sin3 isoform in *S. cerevisiae*, this isoform, along with the HDAC Rpd3, is found in two complexes. RPD3L and RPD3S are similar to Complex I and Complex II of *S. pombe* [50]. In *Xenopus*, the methyl-CpG binding protein 2 (MeCP2) forms a complex with SIN3 and a histone deacetylase [51]. MeCP2 binds to methylated DNA through its methyl-CpG binding domain and recruits the SIN3 complex to promote transcriptional silencing. Co-immunoprecipitation assays performed using oocyte extracts showed that MeCP2 immunoprecipitates with the *Xenopus* Sin3A variant but not the Sin3B variant. In *Drosophila*, the predominant SIN3 isoforms SIN3 187 and SIN3 220 are part of distinct histone-modifying complexes [52]. Both complexes contain a common set of components that include RPD3, SDS3, ING1, Pf1, Arid4B, SAP130, and BRMS1. In addition to these components, the SIN3 220 complex also contains three unique interaction partners, Caf1-p55, dKDM5/LID, and EMSY. The *Drosophila* SIN3 isoforms thus associate with distinct histone-modifying activities. SIN3 187 interacts with a single catalytic enzyme, RPD3, which is an HDAC, whereas SIN3 220 interacts with the deacetylase and the histone demethylase dKDM5/LID. The SIN3 220 isoform has a unique stretch of C-terminal amino acids relative to the other isoforms. Analysis using structural prediction software such as IUPRED and ANCHOR suggests that the unique C-terminal region is unstructured and has potential protein binding affinity [78]. It is possible that the SIN3 220 C-terminus is involved in specialized interactions with the unique complex components, which impact stability of the SIN3 protein and contribute to the flexibility of SIN3 function. These SIN3 complexes could establish distinct histone modification patterns on their target genes, which may be responsible for the unique gene regulatory control performed by the SIN3 isoforms.

**Table 3:** Enzymatic complex components in SIN3 histone-modifying complexes in different species

Organism	Complex	Enzymatic complex component
*S. cerevisiae* [50]	RPD3L	RPD3 (Histone deacetylase)
	RPD3S	RPD3 (Histone deacetylase)
*S. pombe* [49]	Complex I/I’	Clr6 (Histone deacetylase)
	Complex II	Clr6 (Histone deacetylase)
*D. melanogaster* [52, 77]	SIN3 187	RPD3 (Histone deacetylase)
	SIN3 220	RPD3 (Histone deacetylase) dKDM5/LID (Histone demethylase)
*M. musculus* [8–10, 53]	SIN3A	HDAC1, HDAC2 (Histone deacetylase)
	SIN3B	HDAC1, HDAC2 (Histone deacetylase) RBP2 (Histone demethylase)

Mammalian SIN3 isoforms also exhibit differential protein interactions. Interestingly, like the *Drosophila* SIN3 220 isoform, a histone demethylase has a preferential interaction with the mammalian SIN3B protein. In differentiated myotube extracts, RBP2, which is a homologue of dKDM5/LID, co-immunoprecipitated with SIN3B but not with SIN3A [53]. A significant overlap in SIN3B and RBP2 binding on common target genes was observed using high-density tilling arrays in these cells, indicating coordinated binding of these proteins. The SHMP complex, which consists of SIN3B, HDAC1, MRG15, and Pf1, is another example of a distinct set of protein interactions exhibited by mammalian SIN3 proteins [54]. In HeLa cells, the Pf1-SIN3B-containing complex binds to constitutively transcribed genes and regulates their level of expression. These researchers found that endogenous Pf1 preferentially interacts with SIN3B but not with SIN3A in co-immunoprecipitation assays. Additionally, they determined that loss of Pf1 and MRG15 significantly affects the recruitment of SIN3B at these genes, but the level of SIN3A is unaffected, further emphasizing the specificity of interactions within this complex. In another study, however, SIN3A was reported to interact with both Pf1 and MRG15 in HEK293 cells [55]. These data clearly exemplify the versatility of SIN3 proteins in forming distinct complexes in a cell type or context-dependent manner, thereby broadening their scope of regulation of cellular processes.

Like SIN3B, SIN3A also exhibits preferential interactions with chromatin-associated factors. The hormone-sensitive transcriptional corepressor SMRT directly interacted with SIN3A in an in vitro interaction assay, but no detectable interaction was observed between SMRT and SIN3B [56]. SIN3A also interacts with ATPases involved in chromatin remodeling, BRG1 and hBRM [57]. The BRG1 complex consists of SIN3A, HDAC2, and RbAp48, while the hBRM complex contains HDAC1 in addition to these proteins. The role or fate of SIN3B in this specific complex was not reported in this study. In fact, many protein–protein interaction studies have focused on single SIN3 isoforms or did not distinguish between the different SIN3 proteins. A detailed analysis of the diverse protein interaction networks mediated by SIN3 isoforms in different species is lacking. To understand the complete picture of the fine-tuned regulation of gene expression and downstream biological processes by the global transcriptional regulator SIN3, mapping these distinct networks regulated by SIN3 isoforms is critical.

## Differential regulation of biological processes by SIN3 isoforms

As summarized above, in multiple organisms, different SIN3 isoforms interact with a variety of common as well as distinct binding partners. The presence of multiple isoforms, with evolutionarily conserved functional domains capable of unique protein interactions, clearly suggests that these proteins perform non-redundant biological functions. Several studies provide evidence for the specialized roles of SIN3 isoforms. In *Drosophila*, SIN3 isoforms exhibit differential expression during different stages of embryogenesis [34]. In the initial stages of *Drosophila* embryogenesis, equivalent levels of the SIN3 isoforms are observed. The higher molecular weight isoform SIN3 220 gains predominance during stages 12–16 of embryo development and is drastically reduced in stage 17, the final stage of embryogenesis. Conversely, the lower molecular weight isoforms SIN3 187 and SIN3 190 exhibit predominant expression during stage 17. This differential expression pattern of SIN3 isoforms suggests that these proteins possibly target different gene sets and play distinct roles during embryonic development. The study by Dobi et al. [58] analyzing the role of SIN3 in muscle morphogenesis in *Drosophila* embryos provides evidence for the differential role of the SIN3 isoforms during development. Overexpression of SIN3 187 in the mesoderm resulted in severe defects in muscle shape and identity, whereas misexpression of SIN3 220 did not exhibit such effects. These findings suggest that SIN3 187 plays a more predominant role in regulating muscle cell identity genes in the mesoderm. Furthermore, genome-wide recruitment and transcriptome analysis performed in *Drosophila* S2 cells identified genes that are specifically regulated by the SIN3 187 isoform [20]. Interestingly, gene ontology (GO) analysis of SIN3 187-regulated genes (Table 1) shows enrichment for biological processes such as post-embryonic development, metamorphosis, and apoptosis, which is consistent with the observed predominant expression of SIN3 187 during later stages of embryo development.

The mammalian SIN3 proteins are also critical for normal embryonic development. Distinct phenotypic effects are observed upon loss of either SIN3A or SIN3B [14, 59, 60]. The *Sin3a* null mouse embryos survive to embryonic day 3.5 (E3.5) but cannot be detected at E6.5, indicating that SIN3A is essential in early embryo development [14, 59]. Importantly, the presence of the highly related SIN3B protein cannot compensate for the loss of SIN3A. Instead, *Sin3b* null embryos can survive to E15.5, implying that SIN3B function is required in late gestation [60].

Analysis of SIN3 isoform function in specific cell types further emphasizes the differential regulation performed by these proteins. In myoblasts and skeletal muscles, inactivation of *Sin3a* leads to a severe phenotype as compared to loss of *Sin3b* [61]. Mice with a *Sin3a* deletion in the myoblast compartment died within 24 h after birth, while those with deleted *Sin3a* in differentiated skeletal muscles did not survive beyond 2 weeks. Conversely, *Sin3b* deletion in myoblasts and skeletal muscles did not result in any obvious defects in development or survival as compared to control mice. Strikingly, inactivation of both *Sin3a* and *Sin3b* in skeletal muscles led to significantly shorter survival relative to the loss of each individual SIN3 protein. SIN3A and SIN3B also possibly regulate distinct pathways in hematopoietic stem cells (HSCs). *Sin3a* deletion in the bone marrow resulted in a significant loss in the number of HSCs and immediate progenitor cells [62]. In contrast, inactivation of *Sin3b* did not affect HSC viability but instead caused a defect in the differentiation of HSCs into progenitor cells [63].

Mammalian SIN3 proteins also impact pluripotency. While both isoforms function in pluripotent P19 cells, SIN3A exhibits a higher degree of repression of neuronal gene activity as compared to SIN3B [64]. Knockdown of *Sin3a* resulted in decreased expression of REST [repressor element-1 (RE-1) silencing transcription factor] and consequently an increase in the level of neuronal markers, leading to the differentiation of P19 cells into neurogenic cells. *Sin3b* silencing in these cells, however, caused a very small effect on the expression of neuronal markers, and the differentiation into neuronal cells was less efficient relative to *Sin3a* knockdown. These data suggest that SIN3A plays a predominant role in REST-mediated suppression of neuronal differentiation in pluripotent cells. Furthermore, SIN3A is a player in the process of somatic cell reprogramming [45]. Knockdown of *Sin3a* significantly reduced the efficiency of Oct4, Sox2, Klf4, Myc (OSKM)-mediated MEF reprogramming. This study also showed that co-expression of SIN3A with NANOG in partially reprogrammed neural stem cells increased the efficiency of reprogramming more than threefold as compared to NANOG alone. This reprogramming synergy with NANOG was not exhibited by SIN3B, indicating that this function is specific to SIN3A. These examples provide evidence of SIN3 isoform-specific function in regulating developmental cell fate decisions.

Provided the data that SIN3 proteins play critical roles in the regulation of important biological processes, misregulation of SIN3 is implicated in several diseases. The role of SIN3 in cancer has been an area of particular focus. While well studied, the function of SIN3 in cancer cells is still ambiguous, since different investigations attribute either tumor suppressive or oncogenic functions to SIN3 proteins [27, 65]. Interestingly, a recent study showed that the highly related human SIN3 isoforms perform opposing functions in breast cancer metastasis [66]. Loss of *SIN3A* caused a significant increase in the number of invasive colonies in multiple human breast cancer cell lines, MDA-MB-231, MDA-MB-435, and MDA-MB-436. In contrast, *SIN3B* knockdown substantially decreased breast cancer cell invasion and resulted in reduced metastatic potential. Cells with dual knockdown of *SIN3A* and *SIN3B* behaved similar to those with loss of only *SIN3B*. In that same study, the authors performed correlation analysis, investigating SIN3A and SIN3B expression levels in a number of breast cancer subtypes. When all breast cancer subtypes were considered, longer relapse-free survival of patients correlated with high expression of either SIN3A or SIN3B. Analysis of triple-negative breast cancer samples, however, indicated that longer relapse-free survival is correlated with either high SIN3A or low SIN3B expression. These data suggest that there may be functional differences between the SIN3 isoforms in different molecular subtypes of cancer. This study is especially interesting in light of the current interest in SIN3 as a potential therapeutic target [65]. Future efforts should be directed toward a better understanding of the precise mechanism of regulation by individual SIN3 isoforms in different cell types and especially during cancer progression.

## Conclusions

SIN3 was discovered as a transcriptional regulator three decades ago. In subsequent years, a plethora of studies have implicated SIN3 proteins in the regulation of several critical biological processes and revealed a large repertoire of binding partners. Despite this extensive research, we are far from understanding the complete picture of SIN3 regulation. Several pieces of the puzzle are still missing. As discussed above, the SIN3 protein consists of multiple protein interaction domains and hence is considered the scaffold that holds together the SIN3 histone-modifying complex. To the best of our knowledge, however, no study has been conducted to analyze complex integrity upon loss of SIN3. Systematic biochemical analysis of SIN3 complex structure and stability will provide further insight into the scaffolding function of SIN3. Additionally, SIN3 complexes are canonically considered as corepressor complexes that suppress gene expression through the activity of HDACs. This model has been challenged with the acquisition of gene expression and chromatin binding data indicating that SIN3 is likely required for direct activation of a subset of targets. The gene activation function of SIN3 histone-modifying complexes is not at all understood. Genome-wide analysis of histone modification patterns established by the distinct SIN3 complexes at target genes may help us better understand the role of SIN3 in both activation and repression of gene expression.

In this review, we have focused on isoforms of SIN3. There is a single SIN3 protein in the budding yeast, *Saccharomyces cerevisiae*, a single gene that produces multiple isoforms in *Drosophila* and two separate genes that give rise to different isoforms in mammalian cells. Despite the diversity in the number and structure of genes, the SIN3 proteins in different species contain evolutionarily conserved functional domains and form similar histone-modifying complexes. It will be a worthy effort to investigate the evolution of SIN3 proteins and determine whether the presence of multiple isoforms in higher organisms contributes to the functional flexibility of SIN3. The SIN3 complexes are pleiotropic in nature, and this in part contributes to their wide-range of regulation of biologically important processes. Significant efforts must be directed toward identifying the diverse common and unique interaction partners of SIN3 isoforms in different cell types. This will aid in understanding the intricate network of transcriptional regulators and in turn the critical cellular processes that may be impacted upon deregulation of SIN3. There is also a gap in the existing knowledge regarding processes that regulate SIN3 protein expression. Understanding the mechanisms that regulate the global transcriptional regulator SIN3 is crucial, especially since altered levels of SIN3 have been detected in several types of cancer. Furthermore, the SIN3 isoforms may regulate distinct biological pathways in different cell types. It is imperative to carefully dissect the functional differences between SIN3 isoforms and identify gene targets that are differentially regulated. This will prove to be particularly important in designing therapeutics that are targeted for specific cancer subtypes.

In summary, we provide here a few exciting avenues to further our understanding of epigenetic regulation of gene expression by SIN3 complexes. Although a great deal is known about the interactions of SIN3 proteins and the biological processes regulated by them, the current need is to delve deeper into the intricacies of this network. Ascertaining the overlapping and specialized functions of individual SIN3 isoforms will not only unravel novel strategies of gene regulation but will also expand the current repertoire of therapeutic targets.

## Abbreviations

HATs: histone acetyltransferases
HDACs: histone deacetylases
PAH: paired amphipathic helix
HID: histone deacetylase interaction domain
HCR: highly conserved region
HSCs: hematopoietic stem cells
HO: homothallic switching
TOPORS: TOP1 binding arginine/serine-rich protein
MeCP2: methyl-CpG binding protein 2
REST: repressor element-1 (RE-1) silencing transcription factor
